# The SNPs in pre-miRNA are related to the response of capecitabine-based therapy in advanced colon cancer patients

**DOI:** 10.18632/oncotarget.23190

**Published:** 2017-12-11

**Authors:** Yong Mao, Chengda Zou, Fanyi Meng, Jiehong Kong, Weipeng Wang, Dong Hua

**Affiliations:** ^1^ Department of Medical Oncology, Institute of Cancer, Affiliated Hospital of Jiangnan University and The Fourth People's Hospital of Wuxi, Wuxi 214062, China; ^2^ Center for Drug Metabolism and Pharmacokinetics, College of Pharmaceutical Sciences, Soochow University, Suzhou 215123, China; ^3^ Department of Orthopedics, Children's Hospital of Soochow University, Suzhou 215123, China

**Keywords:** colon cancer, capecitabine, polymorphism, microRNA

## Abstract

The single nucleotide polymorphisms (SNPs) in the microRNA precursor (pre-miRNA) may modulate the posttranscriptional regulation of gene expression and explain individual sensitivity to chemotherapy. Here we investigated the correlation between 23 SNPs in the pre-miRNA and the efficacy of capecitabine-based chemotherapy in 274 advanced colon cancer patients. Statistical analysis indicated that much more patients with rs744591 A/C(48.03%), C/C (53.45%) or C allele (49.73%) responded to the chemotherapy than those with the A/A genotype (33.71%). The response rates of rs745666 G/C heterozygous patients (35.25%) and C allele carriers (39.69%) were apparently less than that of the G/G homozygous patients (56.25%). Moreover, three SNPs rs2114358, rs35770269, and rs73239138 were significantly associated with the occurrence of side effects of chemotherapy. The patients with rs2114358 C allele (OR = 2.016) or rs35770269 T allele (OR = 2.299) were much more prone to endure adverse events. However, the incidence of side effect was lower in the patients carrying rs73239138 A allele than those with G/G genotype (OR = 0.500). Our findings demonstrate that genetic variations in pre-miRNA may influence the efficacy of capecitabine-based chemotherapy in advanced colon cancer patients.

## INTRODUCTION

Colorectal carcinoma (CRC), including colon cancer and rectal cancer, is one of the most common gastrointestinal malignancies. The global annual incidence of new CRC cases reaches to about 1.36 million, but decreasing mortality rates have been observed in a large number of countries worldwide [1]. Although the incidence rates of CRC in Asia are lower than those in the United States and Europe, the incidence and mortality rates of CRC have also increased along with the changes of lifestyle and diet in China [2]. At present, CRC screening, reduced prevalence of risk factors, and/or improved treatments have been considered to be effective measures to reduce incidence and mortality rates of CRC.

The treatments for CRC include surgical treatment, chemotherapy, radiotherapy and/or immunotherapy. Surgical treatment is preferred for early CRC with a 5-year survival rate of up to 90%. But for the advanced CRC patients, treatment with chemical drugs is required. Capecitabine, an oral prodrug of 5-fluorouracil, is a first-line chemotherapy drug for the advanced CRC patients [3]. However, the efficacy and occurrence of adverse events are quite different among the patients [4]. Due to the heterogeneity of cancer cells, the cell sensitivity to chemotherapy drugs is quite different, which leads to significant individual response and occurrence of adverse events in patients [5]. Thus, uncovering the genetic mechanism underlying the individual response to chemotherapy is of great significance to improve the efficacy and reduce the eventuation of adverse events.

Single nucleotide polymorphism (SNP), the most common genetic variation, has been validated to contribute a lot to drug response, especially the SNPs in the regulatory genes [6]. MicroRNA (miRNA), a non-coding small RNA consisting of about 22 nucleotides, plays a crucial role in a variety of biological functions, such as cell proliferation, differentiation, development, senescence, and apoptosis [7]. miRNA is first transcripted from genomic DNA to pri-miRNA and then is digested by Drosha enzyme to pre-miRNA, which is transported from nucleus to cytoplasm. In the cytoplasm, the pre-miRNA is cleaved by Dicer enzyme to yield the mature miRNA. The mature miRNA can regulate the translation of target genes by binding to the 3’-UTR of mRNA [8].

More and more studies have provided evidences to demonstrate that the SNPs in the miRNA genes, through impacting the interaction of miRNA with target genes and/or affecting the expression level of mature miRNA, result in dysregulation of target gene and consequent individual drug response and toxicity [9]. Several studies have shown that the miRNA-related SNPs are related to the efficacy and/or occurrence of adverse events of capecitabine-based chemotherapy for CRC patients [10, 11]. For instance, Sclafani *et al*. have shown that the rs4919510 CC genotype in miR-608 is associated with worse outcome in rectal cancer patients treated with neoadjuvant systemic chemotherapy followed by surgery and adjuvant chemotherapy [10]. Meulendijks *et al*. have shown that polymorphisms in the miR-27a genomic region (MIR27A) can be used to improve the predictive value of DPYD variants to identify the risk of severe fluoropyrimidine associated toxicity in cancer patients. The patients carrying a DPYD^+^/MIR27A^+^ genotype are at much higher risk of early severe fluoropyrimidine associated toxicity than those with a DPYD^+^/MIR27A^−^ genotype [11]. Another common polymorphism (rs895819A > G) in MIR27A was found to be associated with a early-onset toxicity in fluoropyrimidine-based chemotherapy in the patients carrying DPYD risk variants [12]. We previously revealed that an SNP rs7911488 T > C in pre-miR-1307 was significantly associated with the efficacy of capecitabine in colon cancer patients. The rs7911488 C allele leads to low-expression of miR-1307-3p, high-expression of TYMS, as well as insensitive to capecitabine chemotherapy [13].

In this study, we selected 32 polymorphisms in pre-miRNA and successfully detected 23 loci in 274 advanced colon cancer patients. The association between the genotypes and the efficacy and incidence of adverse events of chemotherapy were statistically analyzed. The results demonstrate that two polymorphisms are significantly related to the efficacy and three loci contribute to the occurrence of adverse events of chemotherapy.

## RESULTS

### The genotyping results

To investigate the association of SNPs in the pre-miRNA with the response of capecitabine-based chemotherapy, we searched 304 SNPs and their allele frequencies from the online databases NCBI dbSNP (Supplementary Table 1). Among these SNPs, the minor allele frequencies of 32 SNPs were more than 10% (Supplementary Table 1). We then detected these 32 polymorphisms by using MassARRAY SNP Genotyping method. In which, 23 SNPs were successfully detected in 274 advanced colon cancer patients, but the determination of the other 9 SNPs was failed due to potential interaction among primers and/or unspecific amplification. The typical genotyping results of 17 SNPs were showed in Supplementary Figure 1. The results of the other 6 SNPs located in the terminal-loops of pre-miRNAs have been published before [13].

### The correlation between genotypes and the efficacy of capecitabine-based chemotherapy

The correlation between genotypes and the efficacy of capecitabine-based chemotherapy was statistically analyzed and the results were listed in Table 1. The results indicated that rs744591 and rs745666 were significantly related to the response of capecitabine-based chemotherapy in this cohort of advanced colon cancer patients. The response rate of the patients with rs744591 A/C genotype (48.03%; *P* = 0.037) or C/C (53.45%; *P* = 0.018) genotype, or carrying A-allele (49.73%; *P* = 0.013) were obviously much higher than that of the A/A homozygous patients (33.71%). When compared with the G/G homozygous patients, the response rates of rs745666 G/C heterozygous patients (35.25% vs 56.25%; *P* = 0.003) and C-allele carriers (39.69% vs 56.25%; *P* = 0.012) was apparently lower. The response rate of rs745666 G/C heterozygous patients was even less than that of the patients carrying homozygous genotypes GG and CC (35.25% vs 54.07%; *P* = 0.002). There is no relationship between the other 15 SNPs (rs174561, rs670637, rs2043556, rs2114358, rs2289030, rs2663345, rs4919510, rs9913045, rs10061133, rs11614913, rs13299349, rs35770269, rs61992671, rs67106263, and rs73239138) and the efficacy of the capecitabine-based chemotherapy (Supplementary Table 2).

**Table 1:** The association of genotypes with the efficacy of chemotherapy

SNP	Genotype	Efficacy (n)^a^		Response rate (%)	OR (95% CI)^b^	*P*-value^c^
		PD + SD	PR + CR			
rs744591	A/A	59	30	33.71	Reference	1.000
	A/C	66	61	48.03	1.815 (1.044–3.155)	**0.037**
	C/C	27	31	53.45	2.342 (1.193–4.608)	**0.018**
	A/C-C/C	93	92	49.73	1.969 (1.171–3.300)	**0.013**
	A/A-A/C	125	91	42.13	Reference	1.000
	C/C	27	31	53.45	1.639 (0.912–2.941)	0.109
	A/A-C/C	86	61	41.50	Reference	1.000
	A/C	66	61	48.03	1.280 (0.795–2.062)	0.334
rs745666	G/G	35	45	56.25	Reference	1.000
	G/C	90	49	35.25	0.421 (0.241–0.739)	**0.003**
	C/C	27	28	50.91	0.794 (0.399–1.580)	0.598
	G/C-C/C	117	77	39.69	0.508 (0.300–0.861)	**0.012**
	G/G-G/C	125	94	42.92	Reference	1.000
	C/C	27	28	50.91	1.359 (0.754–2.451)	0.369
	G/G-C/C	62	73	54.07	Reference	1.000
	G/C	90	49	35.25	0.463 (0.286–0.751)	**0.002**

^a^ PD, progressive disease; SD, stable disease; PR, partial response; CR, complete response. ^b^ OR, odds ratio; CI, confidence interval. ^c^ The *P* values < 0.05 were in bold.

### The correlation between genotypes and the occurrence of adverse events of capecitabine-based chemotherapy

As shown in Table 2, three SNPs rs2114358, rs35770269, and rs73239138 were significantly associated with the occurrence of side effects of capecitabine-based chemotherapy in the advanced colon cancer patients. As compared with the rs2114358 T/T homozygous patients, the risk of occurring adverse events in those with T/C (OR = 1.876; *P* = 0.019) or C/C (OR = 2.639; *P* = 0.038) genotype or carrying C allele (OR = 2.016; *P* = 0.005) was significantly higher. The patients with rs35770269 A/T genotype (OR = 2.012; *P* = 0.013), T/T genotype (OR = 3.759; *P* = 0.001), or T allele (OR = 2.299; *P* = 0.002) were much more prone to endure adverse events than the A/A homozygous ones. However, the incidence of side effects was lower in the patients with rs73239138 G/A genotype (51.46%; *P* = 0.027) or A/A genotype (48.21%; *P* = 0.021), or carrying A allele (50.31%; *P* = 0.007) than those with G/G genotype (66.96%). No association was observed between the other 14 SNPs (rs174561, rs670637, rs744591, rs745666, rs2043556, rs2289030, rs2663345, rs4919510, rs9913045, rs10061133, rs11614913, rs13299349, rs61992671, and rs67106263) and the incidence of side effects of the capecitabine-based chemotherapy (Supplementary Table 3).

**Table 2:** The association of genotypes with the side effects of chemotherapy

SNP	Genotype	Side effect (n)		Incidence (%)	OR (95% CI)^a^	*P*-value^b^
		No	Yes			
rs2114358	T/T	54	90	62.50	Reference	1.000
	T/C	26	75	74.26	1.876 (1.115–3.165)	**0.019**
	C/C	6	23	79.31	2.639 (1.093–6.369)	**0.038**
	T/C-C/C	32	98	75.38	2.016 (1.238–3.289)	**0.005**
	T/T-T/C	80	165	67.35	Reference	1.000
	C/C	6	23	79.31	2.058 (0.872–4.854)	0.110
	T/T-C/C	60	113	65.32	Reference	1.000
	T/C	26	75	74.26	1.621 (0.978–2.688)	0.076
rs35770269	A/A	34	49	59.04	Reference	1.000
	A/T	43	103	70.55	2.012 (1.164–3.484)	**0.013**
	T/T	9	36	80.00	3.759 (1.698–8.333)	**0.001**
	A/T-T/T	52	139	72.77	2.299 (1.359–3.906)	**0.002**
	A/A-A/T	77	152	66.38	Reference	1.000
	T/T	9	36	80.00	2.404 (1.178–4.902)	**0.020**
	A/A-T/T	43	85	66.41	Reference	1.000
	A/T	43	103	70.55	1.297 (0.803–2.092)	0.329
rs73239138	G/G	32	91	73.98	Reference	1.000
	G/A	35	65	65.00	0.523 (0.302–0.905)	**0.027**
	A/A	19	32	62.75	0.460 (0.239–0.883)	**0.021**
	G/A-A/A	54	97	64.24	0.500 (0.304–0.822)	**0.007**
	G/G-G/A	67	156	69.96	Reference	1.000
	A/A	19	32	62.75	0.630 (0.349–1.136)	0.132
	G/G-A/A	51	123	70.69	Reference	1.000
	G/A	35	65	65.00	0.683 (0.417–1.119)	0.133

^a^ OR, odds ratio; CI, confidence interval. ^b^The *P* values < 0.05 were in bold.

### The regulatory roles of SNPs in the expression of miRNA target genes

The SNPs in the pre-miRNA might through impacting the expression level of mature miRNA and/or the interaction of miRNA:mRNA, result in deregulation of miRNA target genes and play an important role in chemotherapy response. The SNPs rs744591, rs745666, rs2114358, rs35770269, and rs73239138 are located in miR-3196 precursor, miR-3615 precursor, miR-1206 precursor, mature miR-449c, and mature miR-1269a, respectively (Table 3). By using online software TargetScan, we predicted the target genes of miR-3196, miR-3615, miR-1206, miR-449c, and miR-1269a. We found that there were a number of target genes of these miRNAs participated in the fluoropyrimidine pharmacodynamic and pharmacokinetic pathways (Table 3). These findings indicate that these SNPs might through disrupting the expression of the miRNAs and/or the interaction of the miRNAs with the target genes, leading to dysregulation of target genes, contribute to interindividual response and occurrence of adverse events of capecitabine-based chemotherapy. However, the molecular mechanisms are still needed to be further investigated.

**Table 3:** The predicted targets for the polymorphic miRNAs

SNP	miRNA	Location of SNP	Predicted targets ^a^
rs744591	miR-3196	terminal loop	CDA, DHFR, FPGS, SMUG1, UMPS, XRCC3
rs745666	miR-3615	terminal loop	ERCC2, NT5C, TP53
rs2114358	miR-1206	anti-sense	ABCG2, FPGS, NT5C1A, SLC28A1, TYMS, UCK2, XRCC3
rs35770269	miR-449c	mature	CDA, NT5C1A, NT5C3A, SLC22A7, UCK2, UMPS
rs73239138	miR-1269a	mature	NME1, SHMT1, SLC29A1, TP53, UCK1

^a^The target genes are obtained from the Fluoropyrimidine Pathway (PharmGKB: https://www.pharmgkb.org/pathway/PA150653776).

## DISCUSSION

Numerous studies have provided evidences to support that genetic polymorphisms contribute a lot to the interindividual difference in response and toxicity of drug treatments. In this study, we discovered that two SNPs in pre-miRNA (rs744591 and rs745666) were significantly correlated to the response of capecitabine-based chemotherapy in advanced colon cancer patients. The patients with rs745666 G/G genotype (56.25%) had better response to the capecitabine-based chemotherapy. However, the rs744591 A/A homozygous patients (33.71%) had poor response to the capecitabine-based chemotherapy. Furthermore, our results demonstrated that three SNPs (rs2114358, rs35770269, and rs73239138) were apparently associated with the occurrence of adverse events of capecitabine-based chemotherapy in this cohort of patients. (1) The patients with rs2114358 pared with T/C (OR = 1.876), C/C (OR = 2.639) genotype or C allele (OR = 2.016) were much more prone to endure adverse events than the T/T homozygous individuals. (2) The patients with rs35770269 A/T genotype (OR = 2.012), T/T genotype (OR = 3.759), or T allele (OR = 2.299) had significantly higher risk to endure adverse events than the A/A homozygous ones. (3) The incidence of side effects was markedly higher in the patients with rs73239138 G/G genotype (66.96%) than the others.

rs744591 locates in the terminal loop of miR-3196 precursor, which is transcripted from MIR3196, an uncoding gene in the intron of BIRC7 gene. Several studies have demonstrated that miR-3196 plays an important role in tumor suppression. miR-3196 downregulation was observed in basal cell carcinoma [13], breast cancer [14, 15], papillary thyroid carcinoma [16], gastric cardia adenocarcinoma [17], as well as colorectal carcinoma [18]. Furthermore, miR-3196 was identified as a direct target of histone H2AX and shown to inhibit apoptosis in lung cancer cells by targeting p53 upregulated modulator of apoptosis (PUMA) [19]. In this study, we found that rs744591 was in linkage disequilibrium with rs872808, rs1075557, rs1129659, rs2273487, and rs2273492, which were closely related to the expression of BIRC7, the host gene of miR-3196 (Supplementary Table 4). These findings indicate that rs744591 might through dysregulating miR-3196 and its targets, such as CDA, DHFR, FPGS, SMUG1, UMPS, and/or XRCC3 (Table 3), leads to individual response of capecitabine-based chemotherapy in advanced colon cancer patients.

rs2114358 is an SNP in the miR-1206 precursor. Previous studies confirmed that rs2114358 could impact the expression of miR-1206. It was reported that rs2114358 C-allele can significantly increase mature miR-1206 expression in colon cancer cell lines [20]. rs2114358 TT genotype was associated with an increased risk of chronic lymphocytic leukemia by altering the secondary structure and maturation of miR-1206 [21]. Some studies have provided evidences to show that miR-1206 acts as a tumor promoter. miR-1206 expression has been found in increased levels in B cell tumors such as Namalwa and CA-46 [22]. Treatment of the DNA-damaging agent daunorubicin led to increased PVT1 transcripts and miR-1206 expression in tumor cells in a p53-dependent manner [23]. In addition, Gutierrez *et al*. reported that rs2114358 CC genotype was associated with a 4.6-fold increased likelihood of developing methotrexate-induced oral mucositis in a prospective Dutch cohort of pediatric acute lymphoblastic leukemiapatients [24, 25]. In this study, we found that rs2114358 was closely related to the occurrence of adverse events in the advanced colon cancer patients treated by capecitabine, which further expands our understanding of the regulatory role of this polymorphism in tumor.

rs73239138 is a polymorphism located in the mature miR-1269a. Sevaral studied have revealed the regulatory role of both rs73239138 and miR-1269a in carcinogenesis. miR-1269a has been discovered to be up-regulated in tumors and been proved to be a potential biomarker for the prognosis prediction of HCC [26]. The overexpressed miR-1269a promotes cell proliferation in HCC through directly suppressing FOXO1, and functions as an oncomiR in HCC [27]. rs73239138 in miR-1269a has been identified as a protective factor which prevents binding to 3’UTR of SOX6 and there by suppresses tumor growth in HCC [28]. This polymorphism also was found to reduce the tumor suppressor effect of miR-1269a possibly by attenuating the expression level of miR-1269a in HCC cells and overexpression of target genes SPATS2L and LRP6, and promoted the susceptibility to HCC [29]. However, the association of rs73239138 with the response of chemotherapy in cancer patients has not been elucidated. Here, we found rs73239138 might through impacting the regulatory role of miR-1269a in the expression of NME1, SHMT1, SLC29A1, TP53, and/or UCK1, contribute to the occurrence of adverse events in the advanced colon cancer patients reveived capecitabine-based chemotherapy.

rs745666 and rs35770269 are located in the terminal loop of miR-3615 precursor and mature miR-449c, respectively. Studies have confirmed both miR-3615 and miR-449c are primarily involved in tumor pathogenesis. It has been reported that miR-3615 is downregulated in Chinese follicular lymphoma [30]. Previous studies have demonstrated that miR-449c downregulation and c-Myc amplification may be involved in the development of non-small cell lung cancer [31] and osteosarcoma [32]. However, the function of rs745666 and rs35770269 are still largely unknown.

In conclusion, we discovered the association of the miRNA-related SNPs with the efficacy and adverse events of capecitabine-based chemotherapy in the advanced colon cancer patients. Although further investigations are still needed to fully understand the regulatory mechanisms of these miRSNPs, they might be regarded as predictors for the individual response and occurrence of adverse events of capecitabine-based chemotherapy. It might be helpful to provide theoretical basis for individualized treatment of advanced colon cancer.

## MATERIALS AND METHODS

### Patients

The study was approved by the institutional review boards of the Affiliated Hospital of Jiangnan University. All experiments were performed in accordance with the relevant guidelines and regulations of the Affiliated Hospital of Jiangnan University. The patients were recruited and treated as reported before [13]. Briefly, 274 patients with advanced colon cancer (169 male and 105 female; 128 of ≤ 60 years and 146 of > 60 years) were treated with 3 weekly cycles of capecitabine (1,000 mg/m^2^ bid. for 14 days) and oxaliplatin (130 mg/m^2^ on day 1). The patients did not previously receive any chemotherapy or adjuvant chemotherapy. The efficacy of chemotherapy was evaluated every 9 weeks according to RECIST (Response Evaluation Criteria in Solid Tumors, Version 1.0). The responses of the patients to the chemotherapy include 15 of complete response (CR), 107 of partial response (PR), 63 of stable disease (SD), and 89 of progressive disease (PD). The adverse events include 82 nausea and vomiting, 56 myelosuppression, 21 liver dysfunction, 16 diarrhea, 8 neurotoxicity, and 5 others. The doses of capecitabine were reduced in patients with adverse reactions. All patients provided written informed consent before study entry. The genomic DNAs were extracted from the whole blood obtained from the patients before therapy. All clinical results were blinded by genotype.

### Genotyping

The SNPs in the miRNA precursors and their allele frequencies (Supplementary Table 1) were obtained from the online databases miRNASNP 2.0 (www.bioguo.org/miRNASNP2) and NCBI dbSNP (https://www.ncbi.nlm.nih.gov/projects/SNP/), respectively. Thirty two SNPs with minor allele frequencies ≥ 10% were determined by using MassARRAY SNP Genotyping method (SEQUENOM, In.) as described before [33]. First, the DNA fragments containing SNPs were amplified by PCR. Then single-base extensions were carried out at the SNP loci. Thirdly, the single-base extension products were desalted and transferred to the chips for matrix-assisted laser desorption ionization / time of flight / mass spectrometry analysis. Finally, the genotypes were read according to the mass spectrums.

### Statistical analysis

The association between genotypes and the efficacy and occurrence of adverse events of capecitabine-based chemotherapy were assessed by using Chi-square test. The odds ratios (ORs) and 95% confidence intervals (CIs) were obtained using Unconditional Univariate Logistic Regression model. The statistical analyses were done by using SPSS11.5 software and by two analysts independently in a blind fashion. A probability value of less than 0.05 was considered statistically significant.

## SUPPLEMENTARY MATERIALS FIGURES AND TABLES









## References

[1] Torre LA, Bray F, Siegel RL, Ferlay J, Lortet-Tieulent J, Jemal A (2015). Global cancer statistics, 2012. CA Cancer J Clin.

[2] Chen W, Zheng R, Baade PD, Zhang S, Zeng H, Bray F, Jemal A, Yu XQ, He J (2016). Cancer statistics in China, 2015. CA Cancer J Clin.

[3] Thorn CF, Marsh S, Carrillo MW, McLeod HL, Klein TE, Altman RB (2011). PharmGKB summary: fluoropyrimidine pathways. Pharmacogenet Genomics.

[4] Hirsch BR, Zafar SY (2011). Capecitabine in the management of colorectal cancer. Cancer Manag Res.

[5] Punt CJ, Koopman M, Vermeulen L (2017). From tumour heterogeneity to advances in precision treatment of colorectal cancer. Nat Rev Clin Oncol.

[6] Pang GS, Wang J, Wang Z, Lee CG (2009). Predicting potentially functional SNPs in drug-response genes. Pharmacogenomics.

[7] Ebert MS, Sharp PA (2012). Roles for microRNAs in conferring robustness to biological processes. Cell.

[8] Bartel DP (2009). MicroRNAs: target recognition and regulatory functions. Cell.

[9] Rukov JL, Shomron N (2011). MicroRNA pharmacogenomics: post-transcriptional regulation of drug response. Trends Mol Med.

[10] Sclafani F, Chau I, Cunningham D, Lampis A, Hahne JC, Ghidini M, Lote H, Zito D, Tabernero J, Glimelius B, Cervantes A, Begum R, De Castro DG (2016). Sequence variation in mature microRNA-608 and benefit from neo-adjuvant treatment in locally advanced rectal cancer patients. Carcinogenesis.

[11] Meulendijks D, Henricks LM, Amstutz U, Froehlich TK, Largiadèr CR, Beijnen JH, de Boer A, Deenen MJ, Cats A, Schellens JH (2016). Rs895819 in MIR27A improves the predictive value of DPYD variants to identify patients at risk of severe fluoropyrimidine-associated toxicity. Int J Cancer.

[12] Amstutz U, Offer SM, Sistonen J, Joerger M, Diasio RB, Largiadèr CR (2015). Polymorphisms in MIR27A Associated with Early-Onset Toxicity in Fluoropyrimidine-Based Chemotherapy. Clin Cancer Res.

[13] Sand M, Skrygan M, Sand D, Georgas D, Hahn SA, Gambichler T, Altmeyer P, Bechara FG (2012). Expression of microRNAs in basal cell carcinoma. Br J Dermatol.

[14] Wang B, Li J, Sun M, Sun L, Zhang X (2014). miRNA expression in breast cancer varies with lymph node metastasis and other clinicopathologic features. IUBMB Life.

[15] Peña-Chilet M, Martínez MT, Pérez-Fidalgo JA, Peiró-Chova L, Oltra SS, Tormo E, Alonso-Yuste E, Martinez-Delgado B, Eroles P, Climent J, Burgués O, Ferrer-Lozano J, Bosch A (2014). MicroRNA profile in very young women with breast cancer. BMC Cancer.

[16] Qiu ZL, Shen CT, Song HJ, Wei WJ, Luo QY (2015). Differential expression profiling of circulation microRNAs in PTC patients with non-131I and 131I-avid lungs metastases: a pilot study. Nucl Med Biol.

[17] Gao S, Zhou F, Zhao C, Ma Z, Jia R, Liang S, Zhang M, Zhu X, Zhang P, Wang L, Su F, Zhao J, Liu G (2016). Gastric cardia adenocarcinoma microRNA profiling in Chinese patients. Tumour Biol.

[18] Nagy ZB, Bartak BK, Kalmar A, Galamb O, Wichmann B, Dank M, Igaz P, Tulassay Z, Molnár B (2017). Comparison of Circulating miRNAs Expression Alterations in Matched Tissue and Plasma Samples During Colorectal Cancer Progression. Pathol Oncol Res.

[19] Xu C, Zhang L, Duan L, Lu C (2016). MicroRNA-3196 is inhibited by H2AX phosphorylation and attenuates lung cancer cell apoptosis by downregulating PUMA. Oncotarget.

[20] Kim HK, Prokunina-Olsson L, Chanock SJ (2012). Common genetic variants in miR-1206 (8q24.2) and miR-612 (11q13.3) affect biogenesis of mature miRNA forms. PLoS One.

[21] Martin-Guerrero I, Gutierrez-Camino A, Lopez-Lopez E, Bilbao-Aldaiturriaga N, Pombar-Gomez M, Ardanaz M, Garcia-Orad A (2015). Genetic variants in miRNA processing genes and pre-miRNAs are associated with the risk of chronic lymphocytic leukemia. PLoS One.

[22] Huppi K, Volfovsky N, Runfola T, Jones TL, Mackiewicz M, Martin SE, Mushinski JF, Stephens R, Caplen NJ (2008). The identification of microRNAs in a genomically unstable region of human chromosome 8q24. Mol Cancer Res.

[23] Barsotti AM, Beckerman R, Laptenko O, Huppi K, Caplen NJ, Prives C (2012). p53-Dependent induction of PVT1 and miR-1204. J Biol Chem.

[24] López-López E, Gutiérrez-Camino Á, Piñán MA, Sánchez-Toledo J, Uriz JJ, Ballesteros J, García-Miguel P, Navajas A, García-Orad Á (2014). Pharmacogenetics of microRNAs and microRNAs biogenesis machinery in pediatric acute lymphoblastic leukemia. PLoS One.

[25] Gutierrez-Camino A, Oosterom N, den Hoed MA, Lopez-Lopez E, Martin-Guerrero I, Pluijm SM, Pieters R, de Jonge R, Tissing WJ, Heil SG, García-Orad A, van den Heuvel-Eibrink MM (2017). The miR-1206 microRNA variant is associated with methotrexate-induced oral mucositis in pediatric acute lymphoblastic leukemia. Pharmacogenet Genomics.

[26] Gan TQ, Tang RX, He RQ, Dang YW, Xie Y, Chen G (2015). Upregulated MiR-1269 in hepatocellular carcinoma and its clinical significance. Int J Clin Exp Med.

[27] Yang XW, Shen GZ, Cao LQ, Jiang XF, Peng HP, Shen G, Chen D, Xue P (2014). MicroRNA-1269 promotes proliferation in human hepatocellular carcinoma via downregulation of FOXO1. BMC Cancer.

[28] Xiong G, Wang Y, Ding Q, Yang L (2015). Hsa-mir-1269 genetic variant contributes to hepatocellular carcinoma susceptibility through affecting SOX6. Am J Transl Res.

[29] Min P, Li W, Zeng D, Ma Y, Xu D, Zheng W, Tang F, Chen J, Shi J, Hu H, Wang J, Yang D, Liu J (2017). A single nucleotide variant in microRNA-1269a promotes the occurrence and process of hepatocellular carcinoma by targeting to oncogenes SPATS2L and LRP6. Bull Cancer.

[30] Pan Y, Guo Y, Luo Y, Li H, Xu Y (2016). MicroRNA expression profiling of Chinese follicular lymphoma by microarray: A preliminary study. Int Immunopharmacol.

[31] Miao LJ, Huang SF, Sun ZT, Gao ZY, Zhang RX, Liu Y, Wang J (2013). MiR-449c targets c-Myc and inhibits NSCLC cell progression. FEBS Lett.

[32] Li Q, Li H, Zhao X, Wang B, Zhang L, Zhang C, Zhang F (2017). DNA Methylation Mediated Downregulation of miR-449c Controls Osteosarcoma Cell Cycle Progression by Directly Targeting Oncogene c-Myc. Int J Biol Sci.

[33] Tang R, Qi Q, Wu R, Zhou X, Wu D, Zhou H, Mao Y, Li R, Liu C, Wang L, Chen W, Hua D, Zhang H, Wang W (2015). The polymorphic terminal-loop of pre-miR-1307 binding with MBNL1 contributes to colorectal carcinogenesis via interference with Dicer1 recruitment. Carcinogenesis.

