# Supplementary material for: The SNPs in pre-miRNA are related to the response of capecitabine-based therapy in advanced colon cancer patients
Source: Oncotarget. 2017 Dec 11;9(6):6793–9. doi: 10.18632/oncotarget.23190 (PMC5805515; doi:10.18632/oncotarget.23190)
Supplement: Supplementary file 1 [file oncotarget-09-6793-s001.pdf]

The SNPs in pre-miRNA are related to the response of capecitabine-based therapy in advanced colon cancer patients

SUPPLEMENTARY MATERIALS

Supplementary Table 1: The information of SNPs in the microRNA precursor. See Supplementary Table 1

Supplementary Table 2: The association of genotypes with the efficacy of chemotherapy. See Supplementary Table 2

Supplementary Table 3: The association of genotypes with the side effects of chemotherapy. See Supplementary Table 3

Supplementary Table 4: The SNPs in linkage disequilibrium with rs744591

miRNA gene	Host gene	LD SNP[a]	r^2	eQTL Qvalue[b]
MIR3196	BIRC7	rs2273487	0.99	0.001398
		rs1075557	0.99	0.013633
		rs1129659	0.83	0.029148
		rs2273492	0.96	0.001838
		rs872808	0.83	0.010002

[a]LD, linkage disequilibrium.[b]The data from seeQTL (http://www.bios.unc.edu/research/genomic_software/seeQTL/).

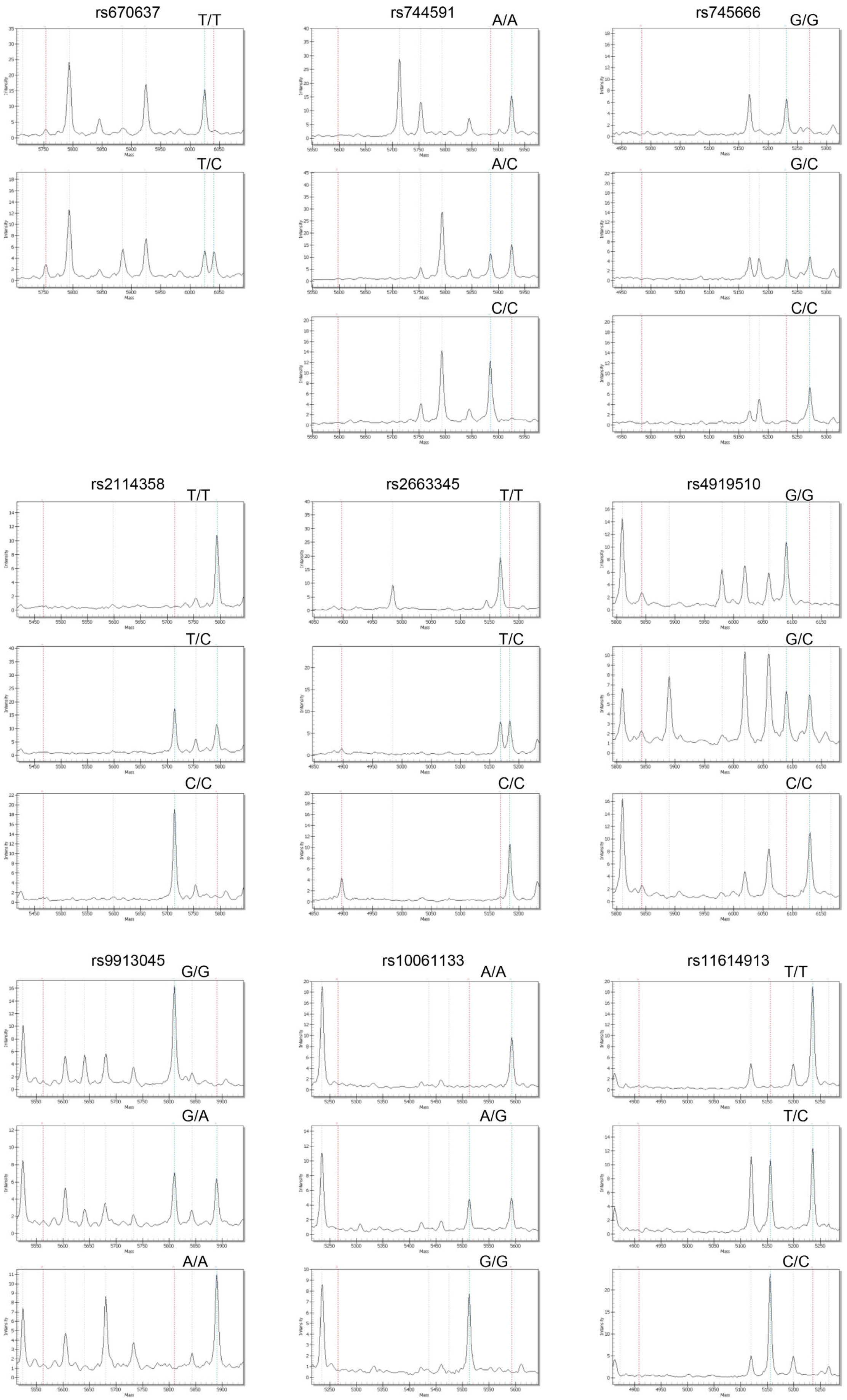
rs670637
T/T
T/C
rs744591
A/A
A/C
C/C
rs745666
G/G
G/C
C/C
rs2114358
T/T
T/C
C/C
rs2663345
T/T
T/C
C/C
rs4919510
G/G
G/C
C/C
rs9913045
G/G
G/A
A/A
rs10061133
A/A
A/G
G/G
rs11614913
T/T
T/C
C/C

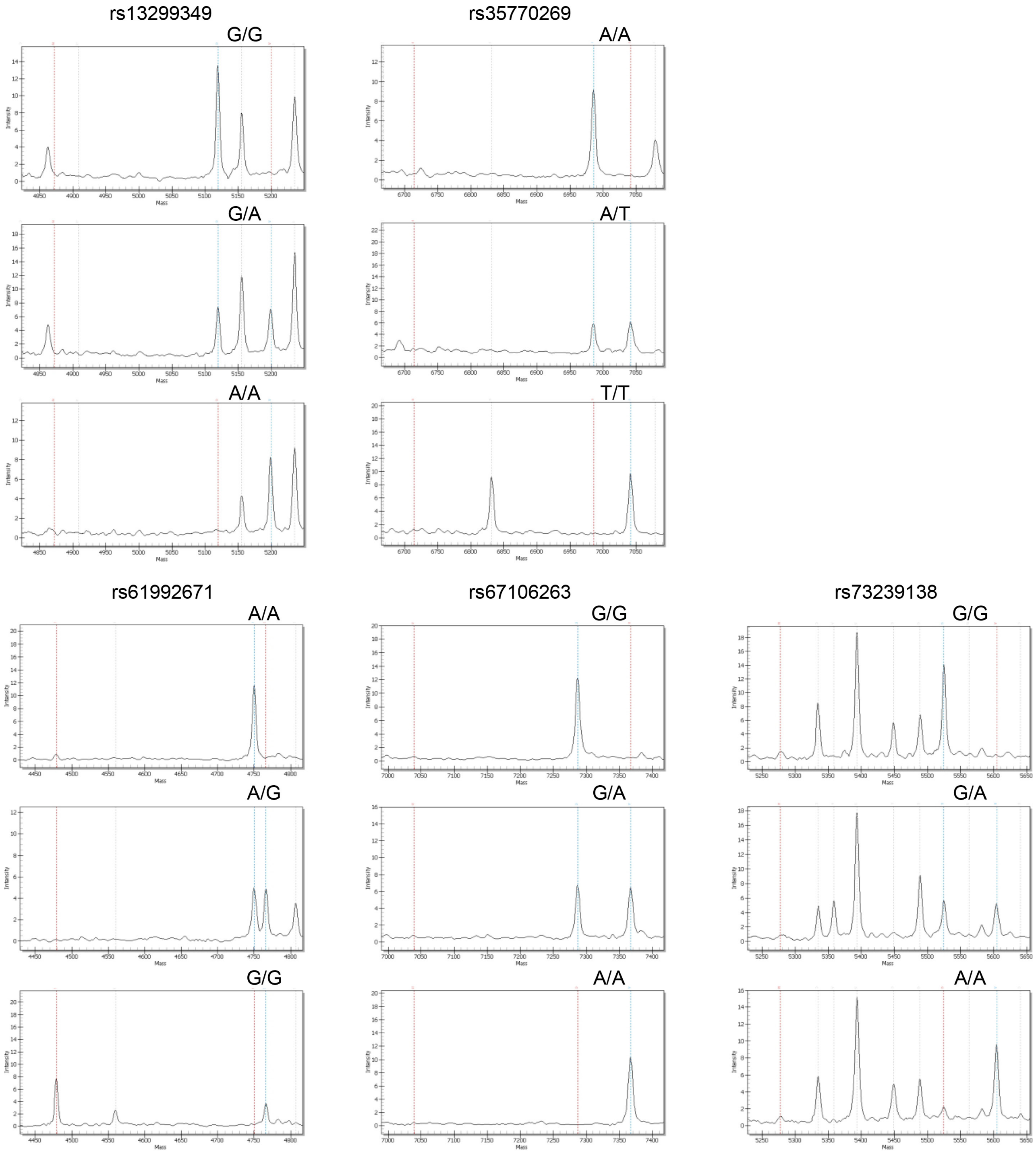

Supplementary Figure 1: The typical genotyping results of SNPs.
