# Supplementary material for: The SNPs in pre-miRNA are related to the response of capecitabine-based therapy in advanced colon cancer patients
Source: Oncotarget. 2017 Dec 11;9(6):6793–9. doi: 10.18632/oncotarget.23190 (PMC5805515; doi:10.18632/oncotarget.23190)
Supplement: Supplementary file 2 [file oncotarget-09-6793-s002.docx]

**Supplementary Table 1** The information of SNPs in the microRNA precursor

| **Location** | **dbSNP ID** | **Allele** | **miRNA** | **MAF**^a^ |
| --- | --- | --- | --- | --- |
| in_seed | rs2910164 | C/G | hsa-mir-146a | **0.3814** |
| in_seed | rs66683138 | G/A | hsa-mir-3622a/b | **0.2935** |
| in_seed | rs12402181 | G/A | hsa-mir-3117 | **0.2665** |
| in_seed | rs35770269 | U/A | hsa-mir-449c | **0.2491** |
| in_seed | rs28599926 | G/A | hsa-mir-1268 | **0.1941** |
| in_seed | rs3746444 | A/G | hsa-mir-499 | **0.1809** |
| in_seed | rs12416605 | G/A | hsa-mir-938 | **0.1529** |
| in_seed | rs62376935 | G/A | hsa-mir-585 | 0.0994 |
| in_seed | rs2620381 | U/G | hsa-mir-627 | 0.0769 |
| in_seed | rs78825966 | C/U | hsa-mir-557 | 0.0595 |
| in_seed | rs9745376 | G/A | hsa-mir-662 | 0.0499 |
| in_seed | rs12220909 | C/G | hsa-mir-4293 | 0.0403 |
| in_seed | rs11973069 | C/U | hsa-mir-4284 | 0.0284 |
| in_seed | rs74647838 | C/U | hsa-mir-1302-1 | 0.0252 |
| in_seed | rs113297757 | G/A | hsa-mir-3196 | 0.0224 |
| in_seed | rs73721294 | C/U | hsa-mir-593 | 0.0197 |
| in_seed | rs114399468 | G/A | hsa-mir-4322 | 0.0188 |
| in_seed | rs79759099 | U/C | hsa-mir-1304 | 0.0174 |
| in_seed | rs74743733 | G/A | hsa-mir-4257 | 0.0114 |
| in_seed | rs12081872 | C/U | hsa-mir-3124 | 0.0105 |
| in_seed | rs116476604 | U/C | hsa-mir-466 | 0.006 |
| in_seed | rs79924817 | A/G | hsa-mir-936 | 0.005 |
| in_seed | rs41292412 | C/U | hsa-mir-122 | 0.0046 |
| in_seed | rs113212828 | A/G | hsa-mir-605 | 0.0041 |
| in_seed | rs73602910 | G/A | hsa-mir-518d | 0.0027 |
| in_seed | rs76084273 | C/A | hsa-mir-3910-1 | 0.0018 |
| in_seed | rs77809319 | A/G | hsa-mir-33a | 0.0009 |
| in_seed | rs78902025 | U/G | hsa-mir-642a | 0.0005 |
| in_seed | rs111835650 | U/C | hsa-mir-411 | NA |
| in_seed | rs112072631 | A/C | hsa-mir-3610 | NA |
| in_seed | rs112977728 | C/U | hsa-mir-3615 | NA |
| in_seed | rs113054794 | G/U | hsa-mir-221 | NA |
| in_seed | rs113098367 | -/A | hsa-mir-3161 | NA |
| in_seed | rs113283070 | G/C | hsa-mir-941-1 | NA |
| in_seed | rs113672516 | G/A | hsa-mir-941-3 | NA |
| in_seed | rs11382316 | -/A | hsa-mir-3161 | NA |
| in_seed | rs11488501 | G/A | hsa-mir-3118-3 | NA |
| in_seed | rs12159555 | C/G | hsa-mir-3618 | NA |
| in_seed | rs12884005 | G/A | hsa-mir-431 | NA |
| in_seed | rs12975333 | G/U | hsa-mir-125a | NA |
| in_seed | rs34059726 | G/U | hsa-mir-124-3 | NA |
| in_seed | rs34381260 | -/A | hsa-mir-1276 | NA |
| in_seed | rs34416818 | -/A | hsa-mir-518e | NA |
| in_seed | rs35027589 | -/G | hsa-mir-513a-1 | NA |
| in_seed | rs35544770 | G/A | hsa-mir-941-4 | NA |
| in_seed | rs4636784 | G/C | hsa-mir-4305 | NA |
| in_seed | rs61786895 | G/A | hsa-mir-3118-2 | NA |
| in_seed | rs61991156 | A/G | hsa-mir-379 | NA |
| in_seed | rs7167371 | C/G | hsa-mir-3118-4 | NA |
| in_seed | rs73024232 | C/U | hsa-mir-3939 | NA |
| in_seed | rs74177813 | C/- | hsa-mir-518e | NA |
| in_seed | rs74177814 | -/G | hsa-mir-518e | NA |
| in_seed | rs75823810 | G/A | hsa-mir-3939 | NA |
| in_seed | rs76608449 | C/G | hsa-mir-3939 | NA |
| in_seed | rs76857625 | U/C | hsa-mir-1304 | NA |
| in_mature | rs9913045 | C/U | hsa-mir-548h-3 | **0.4107** |
| in_mature | rs2241347 | G/A | hsa-mir-3130-1/2 | **0.402** |
| in_mature | rs11614913 | C/U | hsa-mir-196a-2 | **0.3878** |
| in_mature | rs73239138 | G/A | hsa-mir-1269 | **0.3842** |
| in_mature | rs4919510 | C/G | hsa-mir-608 | **0.3466** |
| in_mature | rs61992671 | A/G | hsa-mir-412 | **0.2344** |
| in_mature | rs13299349 | G/A | hsa-mir-3152 | **0.2262** |
| in_mature | rs67106263 | G/A | hsa-mir-3144 | **0.2179** |
| in_mature | rs6513497 | U/G | hsa-mir-646 | **0.1859** |
| in_mature | rs10061133 | U/C | hsa-mir-449b | **0.1268** |
| in_mature | rs11048315 | C/U | hsa-mir-4302 | 0.0994 |
| in_mature | rs6841938 | C/U | hsa-mir-1255b-1 | 0.0929 |
| in_mature | rs61388742 | U/C | hsa-mir-596 | 0.0792 |
| in_mature | rs36092315 | A/- | hsa-mir-509-2 | 0.0597 |
| in_mature | rs59878596 | G/A | hsa-mir-1322 | 0.0572 |
| in_mature | rs78212770 | G/C | hsa-mir-629 | 0.0536 |
| in_mature | rs79402775 | C/U | hsa-mir-933 | 0.049 |
| in_mature | rs13447640 | C/U | hsa-mir-548l | 0.0403 |
| in_mature | rs75330474 | C/U | hsa-mir-323b | 0.0298 |
| in_mature | rs6971711 | C/U | hsa-mir-590 | 0.0275 |
| in_mature | rs78861479 | G/A | hsa-mir-516b-1 | 0.0234 |
| in_mature | rs79957895 | G/A | hsa-mir-3159 | 0.0211 |
| in_mature | rs9589207 | G/A | hsa-mir-92a-1 | 0.0211 |
| in_mature | rs115772313 | C/U | hsa-mir-3130-1/2 | 0.0183 |
| in_mature | rs56660112 | G/C | hsa-mir-3198 | 0.0179 |
| in_mature | rs72631820 | A/G | hsa-mir-339 | 0.0096 |
| in_mature | rs116535054 | G/A | hsa-mir-1274a | 0.0092 |
| in_mature | rs112489955 | G/A | hsa-mir-501 | 0.0078 |
| in_mature | rs112032363 | C/U | hsa-mir-1231 | 0.0078 |
| in_mature | rs116338160 | G/A | hsa-mir-1231 | 0.0078 |
| in_mature | rs73251987 | G/C | hsa-mir-624 | 0.0078 |
| in_mature | rs111525499 | C/U | hsa-mir-3663 | 0.0073 |
| in_mature | rs111899904 | G/A | hsa-mir-629 | 0.0064 |
| in_mature | rs112056940 | C/U | hsa-mir-597 | 0.0064 |
| in_mature | rs115477019 | U/G | hsa-mir-4327 | 0.006 |
| in_mature | rs73735310 | U/C | hsa-mir-586 | 0.006 |
| in_mature | rs114318553 | A/C | hsa-mir-302b | 0.0046 |
| in_mature | rs117258475 | C/U | hsa-mir-296 | 0.0046 |
| in_mature | rs115256251 | A/G | hsa-mir-1252 | 0.0041 |
| in_mature | rs117380662 | G/C | hsa-mir-323b | 0.0041 |
| in_mature | rs77473380 | C/U | hsa-mir-944 | 0.0041 |
| in_mature | rs73772915 | C/G | hsa-mir-2277 | 0.0037 |
| in_mature | rs113256801 | G/C | hsa-mir-664 | 0.0027 |
| in_mature | rs116796400 | A/G | hsa-mir-519d | 0.0027 |
| in_mature | rs115780816 | C/U | hsa-mir-4312 | 0.0018 |
| in_mature | rs78547906 | C/U | hsa-mir-28 | 0.0018 |
| in_mature | rs113384898 | C/U | hsa-mir-548i-2 | 0.0009 |
| in_mature | rs41286570 | G/A | hsa-mir-154 | 0.0009 |
| in_mature | rs114530801 | C/U | hsa-mir-2110 | 0.0005 |
| in_mature | rs10094194 | G/C | hsa-mir-3610 | NA |
| in_mature | rs10655902 | -/C | hsa-mir-4274 | NA |
| in_mature | rs111252952 | -/U | hsa-mir-1273c | NA |
| in_mature | rs111329103 | G/A | hsa-mir-511-1 | NA |
| in_mature | rs111336920 | A/G | hsa-mir-651 | NA |
| in_mature | rs111366342 | A/G | hsa-mir-140 | NA |
| in_mature | rs111479365 | G/A | hsa-mir-941-1 | NA |
| in_mature | rs111586745 | G/A | hsa-mir-941-4 | NA |
| in_mature | rs111664333 | G/A | hsa-mir-642a/b | NA |
| in_mature | rs111715115 | C/U | hsa-mir-1539 | NA |
| in_mature | rs111726405 | A/G | hsa-mir-640 | NA |
| in_mature | rs111737188 | C/U | hsa-mir-3183 | NA |
| in_mature | rs111745142 | -/GU | hsa-mir-520h | NA |
| in_mature | rs111993072 | G/A | hsa-mir-941-3 | NA |
| in_mature | rs112062096 | U/C | hsa-mir-204 | NA |
| in_mature | rs112302475 | A/C | hsa-mir-383 | NA |
| in_mature | rs112314115 | -/CCA | hsa-mir-4274 | NA |
| in_mature | rs112435318 | A/C | hsa-mir-3605 | NA |
| in_mature | rs112495387 | G/A | hsa-mir-523 | NA |
| in_mature | rs112508646 | G/A | hsa-mir-1202 | NA |
| in_mature | rs112544913 | C/U | hsa-mir-4269 | NA |
| in_mature | rs112636147 | U/C | hsa-mir-297 | NA |
| in_mature | rs112850228 | U/C | hsa-mir-888 | NA |
| in_mature | rs112880289 | C/U | hsa-mir-646 | NA |
| in_mature | rs112982830 | A/G | hsa-mir-485 | NA |
| in_mature | rs113220875 | G/C | hsa-mir-4258 | NA |
| in_mature | rs113301642 | G/A | hsa-mir-1537 | NA |
| in_mature | rs113384782 | G/A | hsa-mir-3667 | NA |
| in_mature | rs113642938 | A/G | hsa-mir-4296 | NA |
| in_mature | rs113698386 | C/G | hsa-mir-941-1 | NA |
| in_mature | rs113749278 | U/C | hsa-mir-30c-2 | NA |
| in_mature | rs12625454 | C/G | hsa-mir-941-4 | NA |
| in_mature | rs12731294 | C/A | hsa-mir-4254 | NA |
| in_mature | rs12759620 | G/C | hsa-mir-92b | NA |
| in_mature | rs12894182 | C/A | hsa-mir-625 | NA |
| in_mature | rs13276615 | C/A | hsa-mir-3622a/b | NA |
| in_mature | rs1694089 | U/G | hsa-mir-581 | NA |
| in_mature | rs1886009 | U/C | hsa-mir-3195 | NA |
| in_mature | rs2273488 | C/U | hsa-mir-3196 | NA |
| in_mature | rs2397267 | G/A | hsa-mir-548u | NA |
| in_mature | rs2754157 | A/U | hsa-mir-208b | NA |
| in_mature | rs28632138 | A/C | hsa-mir-568 | NA |
| in_mature | rs33977954 | -/A | hsa-mir-3125 | NA |
| in_mature | rs34604519 | C/G | hsa-mir-941-3 | NA |
| in_mature | rs34610391 | -/A | hsa-mir-606 | NA |
| in_mature | rs34874675 | G/- | hsa-mir-3909 | NA |
| in_mature | rs34887287 | -/U | hsa-mir-3912 | NA |
| in_mature | rs35245133 | -/C | hsa-mir-4274 | NA |
| in_mature | rs35301225 | G/U | hsa-mir-34a | NA |
| in_mature | rs35356504 | C/- | hsa-mir-940 | NA |
| in_mature | rs36100620 | -/G | hsa-mir-1321 | NA |
| in_mature | rs3982072 | A/G | hsa-mir-4252 | NA |
| in_mature | rs3982073 | A/G | hsa-mir-4252 | NA |
| in_mature | rs41274221 | G/A | hsa-mir-25 | NA |
| in_mature | rs41286566 | C/U | hsa-mir-299 | NA |
| in_mature | rs4676066 | G/A | hsa-mir-4265 | NA |
| in_mature | rs60197191 | -/GGCAGCUUUUCAAAGAGCUUU | hsa-mir-3928 | NA |
| in_mature | rs66511565 | -/AC | hsa-mir-4274 | NA |
| in_mature | rs71483239 | G/C | hsa-mir-3689b | NA |
| in_mature | rs71603032 | U/G | hsa-mir-576 | NA |
| in_mature | rs72358191 | -/GAGCUUCGGCAGCUUUUCAAA | hsa-mir-3928 | NA |
| in_mature | rs7255628 | G/C | hsa-mir-520c | NA |
| in_mature | rs72631816 | U/A | hsa-mir-105-2 | NA |
| in_mature | rs72631818 | G/A | hsa-mir-379 | NA |
| in_mature | rs72631821 | C/U | hsa-mir-92a-1 | NA |
| in_mature | rs72647784 | GA/- | hsa-mir-3175 | NA |
| in_mature | rs74878365 | C/A | hsa-mir-148b | NA |
| in_mature | rs76578598 | U/C | hsa-mir-3689b | NA |
| in_mature | rs76759855 | A/G | hsa-mir-605 | NA |
| in_mature | rs77767763 | G/C | hsa-mir-3924 | NA |
| in_mature | rs77814495 | U/C | hsa-mir-1200 | NA |
| in_mature | rs79202905 | G/A | hsa-mir-1911 | NA |
| in_mature | rs79438120 | U/C | hsa-mir-3689b | NA |
| in_mature | rs80166589 | -/C | hsa-mir-3199-1 | NA |
| in_mature | rs810917 | G/A | hsa-mir-581 | NA |
| in_loop | rs2663345 | U/C | hsa-mir-3183 | **0.484** |
| in_loop | rs744591 | C/A | hsa-mir-3196 | **0.4675** |
| in_loop | rs7207008 | U/A | hsa-mir-2117 | **0.429** |
| in_loop | rs745666 | G/C | hsa-mir-3615 | **0.3961** |
| in_loop | rs895819 | A/C | hsa-mir-27a | **0.3581** |
| in_loop | rs6787734 | C/U | hsa-mir-3135a | **0.3388** |
| in_loop | rs2114358 | G/A | hsa-mir-1206 | **0.299** |
| in_loop | rs318039 | C/U | hsa-mir-1274a | **0.2802** |
| in_loop | rs7911488 | U/C | hsa-mir-1307 | **0.2669** |
| in_loop | rs61938575 | G/A | hsa-mir-3922 | **0.2523** |
| in_loop | rs58450758 | C/U | hsa-mir-559 | **0.2001** |
| in_loop | rs670637 | U/C | hsa-mir-3167 | **0.1877** |
| in_loop | rs67339585 | U/C | hsa-mir-3910-1 | **0.1722** |
| in_loop | rs6977967 | U/C | hsa-mir-3683 | **0.1708** |
| in_loop | rs12803915 | G/A | hsa-mir-612 | **0.1658** |
| in_loop | rs2070960 | C/U | hsa-mir-3620 | 0.0998 |
| in_loop | rs11259096 | U/C | hsa-mir-1265 | 0.0879 |
| in_loop | rs58834075 | C/U | hsa-mir-656 | 0.0737 |
| in_loop | rs17797090 | G/A | hsa-mir-3652 | 0.0636 |
| in_loop | rs11873400 | A/G | hsa-mir-4318 | 0.0481 |
| in_loop | rs17091403 | G/A | hsa-mir-2110 | 0.0458 |
| in_loop | rs11020790 | G/A | hsa-mir-548l | 0.0412 |
| in_loop | rs2291418 | C/U | hsa-mir-1229 | 0.0288 |
| in_loop | rs78240175 | G/A | hsa-mir-3123 | 0.027 |
| in_loop | rs115216563 | U/G | hsa-mir-1289-2 | 0.0247 |
| in_loop | rs60861880 | A/G | hsa-mir-3673 | 0.0238 |
| in_loop | rs114814977 | G/U | hsa-mir-922 | 0.0165 |
| in_loop | rs111906529 | U/C | hsa-mir-411 | 0.016 |
| in_loop | rs5965990 | C/U | hsa-mir-891a | 0.0145 |
| in_loop | rs58602811 | A/C | hsa-mir-3119-1 | 0.0137 |
| in_loop | rs73466882 | A/U | hsa-mir-3161 | 0.0137 |
| in_loop | rs114803590 | U/C | hsa-mir-559 | 0.0119 |
| in_loop | rs117767519 | U/G | hsa-mir-4318 | 0.0114 |
| in_loop | rs75102645 | C/U | hsa-mir-3660 | 0.011 |
| in_loop | rs11671784 | C/U | hsa-mir-27a | 0.0105 |
| in_loop | rs79922312 | U/C | hsa-mir-4270 | 0.0092 |
| in_loop | rs78860350 | U/A | hsa-mir-4309 | 0.0082 |
| in_loop | rs72631826 | U/C | hsa-mir-16-1 | 0.0073 |
| in_loop | rs76156362 | U/A | hsa-mir-1275 | 0.0069 |
| in_loop | rs111803974 | C/U | hsa-mir-3908 | 0.0064 |
| in_loop | rs41280052 | G/U | hsa-mir-184 | 0.006 |
| in_loop | rs112439044 | C/U | hsa-mir-30e | 0.0046 |
| in_loop | rs117428639 | C/U | hsa-mir-2113 | 0.0046 |
| in_loop | rs72631824 | C/U | hsa-mir-93 | 0.0046 |
| in_loop | rs72631833 | G/U | hsa-mir-183 | 0.0046 |
| in_loop | rs72631822 | G/A | hsa-mir-130b | 0.0037 |
| in_loop | rs80245767 | A/C | hsa-mir-4286 | 0.0037 |
| in_loop | rs72631827 | G/U | hsa-mir-106b | 0.0027 |
| in_loop | rs74513330 | U/C | hsa-mir-4317 | 0.0027 |
| in_loop | rs75310044 | U/G | hsa-mir-1289-2 | 0.0027 |
| in_loop | rs41274239 | U/C | hsa-mir-96 | 0.0023 |
| in_loop | rs6111978 | U/C | hsa-mir-3192 | 0.0018 |
| in_loop | rs114965408 | C/U | hsa-mir-1272 | 0.0014 |
| in_loop | rs117420576 | C/A | hsa-mir-548o | 0.0014 |
| in_loop | rs76749295 | C/U | hsa-mir-3667 | 0.0009 |
| in_loop | rs112037539 | G/A | hsa-mir-3158-1/2 | 0.0005 |
| in_loop | rs115827677 | G/A | hsa-mir-379 | 0.0005 |
| in_loop | rs72631823 | G/A | hsa-mir-34a | 0.0005 |
| in_loop | rs10549054 | UA/- | hsa-mir-620 | NA |
| in_loop | rs10670323 | -/AAAGA | hsa-mir-516b-2 | NA |
| in_loop | rs111606748 | C/U | hsa-mir-596 | NA |
| in_loop | rs111718468 | U/C | hsa-mir-141 | NA |
| in_loop | rs112205797 | C/U | hsa-mir-941-4 | NA |
| in_loop | rs112257111 | C/U | hsa-mir-939 | NA |
| in_loop | rs112304609 | UU/- | hsa-mir-3938 | NA |
| in_loop | rs11269 | C/A | hsa-mir-1282 | NA |
| in_loop | rs112752440 | G/C | hsa-mir-4258 | NA |
| in_loop | rs113294256 | AU/- | hsa-mir-3939 | NA |
| in_loop | rs113322127 | U/C | hsa-mir-135a-2 | NA |
| in_loop | rs113328518 | AUAU/- | hsa-mir-620 | NA |
| in_loop | rs113464681 | UGU/- | hsa-mir-550a/b-2 | NA |
| in_loop | rs113568246 | G/A | hsa-mir-1470 | NA |
| in_loop | rs113626871 | C/A | hsa-mir-3689b | NA |
| in_loop | rs113909793 | G/A | hsa-mir-1237 | NA |
| in_loop | rs12200860 | A/G | hsa-mir-3939 | NA |
| in_loop | rs1814008 | G/A | hsa-mir-3118-4 | NA |
| in_loop | rs2561251 | G/A | hsa-mir-521-1 | NA |
| in_loop | rs2855372 | G/U | hsa-mir-3180-5 | NA |
| in_loop | rs28575325 | G/A | hsa-mir-3177 | NA |
| in_loop | rs3043743 | UA/- | hsa-mir-620 | NA |
| in_loop | rs33953969 | -/AAAGA | hsa-mir-516b-2 | NA |
| in_loop | rs33982250 | A/- | hsa-mir-1303 | NA |
| in_loop | rs347881 | A/G | hsa-mir-1233-1/2 | NA |
| in_loop | rs347882 | G/C | hsa-mir-1233-1/2 | NA |
| in_loop | rs34889453 | A/- | hsa-mir-1303 | NA |
| in_loop | rs34913856 | G/- | hsa-mir-1260b | NA |
| in_loop | rs34952329 | -/C | hsa-mir-223 | NA |
| in_loop | rs35180578 | -/G | hsa-mir-4259 | NA |
| in_loop | rs35679521 | C/U | hsa-mir-3689b | NA |
| in_loop | rs41281222 | G/A | hsa-mir-183 | NA |
| in_loop | rs41510649 | U/C | hsa-mir-3188 | NA |
| in_loop | rs45556632 | C/G | hsa-mir-639 | NA |
| in_loop | rs55835744 | C/U | hsa-mir-3714 | NA |
| in_loop | rs55921050 | G/A | hsa-mir-3673 | NA |
| in_loop | rs57824231 | AU/- | hsa-mir-3673 | NA |
| in_loop | rs5801168 | AU/- | hsa-mir-620 | NA |
| in_loop | rs59684995 | U/A | hsa-mir-3938 | NA |
| in_loop | rs61953551 | G/U | hsa-mir-3908 | NA |
| in_loop | rs62165009 | U/C | hsa-mir-663b | NA |
| in_loop | rs62555121 | A/U | hsa-mir-1299 | NA |
| in_loop | rs62747560 | GGGGUGG/- | hsa-mir-3652 | NA |
| in_loop | rs71297276 | AUUACUUUCA/- | hsa-mir-548a-3 | NA |
| in_loop | rs71312743 | G/U | hsa-mir-1286 | NA |
| in_loop | rs71440517 | GGGGUGG/- | hsa-mir-3652 | NA |
| in_loop | rs71528599 | U/C | hsa-mir-550a-1 | NA |
| in_loop | rs72631825 | G/A | hsa-mir-222 | NA |
| in_loop | rs73159662 | C/U | hsa-mir-96 | NA |
| in_loop | rs7363975 | A/C | hsa-mir-3196 | NA |
| in_loop | rs74273836 | UUACU/- | hsa-mir-548h-4 | NA |
| in_loop | rs74446573 | U/G | hsa-mir-3908 | NA |
| in_loop | rs74826059 | U/C | hsa-mir-320d-1 | NA |
| in_loop | rs74853538 | U/G | hsa-mir-663b | NA |
| in_loop | rs75245503 | C/G | hsa-mir-663b | NA |
| in_loop | rs75538180 | A/U | hsa-mir-1303 | NA |
| in_loop | rs76132421 | U/C | hsa-mir-3118-1 | NA |
| in_loop | rs77703604 | G/U | hsa-mir-620 | NA |
| in_loop | rs78327226 | G/A | hsa-mir-663b | NA |
| in_loop | rs78666598 | U/A | hsa-mir-1287 | NA |
| in_loop | rs78681286 | U/C | hsa-mir-3180-4 | NA |
| in_loop | rs788517 | G/A | hsa-mir-581 | NA |
| in_loop | rs79594127 | U/C | hsa-mir-3118-2 | NA |
| in_loop | rs79965448 | G/A | hsa-mir-1299 | NA |
| in_loop | rs80176156 | C/G | hsa-mir-3605 | NA |
| in_loop | rs80268200 | A/G | hsa-mir-525 | NA |
| in_loop | rs74864340 | C/U | hsa-mir-550a-1 | NA |

^a^ MAF, minor allele frequency generated by the 1000 Genomes Project; NA, not variable; the MAF≥0.1 were in bold.
