# Supplementary material for: The SNPs in pre-miRNA are related to the response of capecitabine-based therapy in advanced colon cancer patients
Source: Oncotarget. 2017 Dec 11;9(6):6793–9. doi: 10.18632/oncotarget.23190 (PMC5805515; doi:10.18632/oncotarget.23190)
Supplement: Supplementary file 3 [file oncotarget-09-6793-s003.docx]

**Supplementary Table 2** The association of genotypes with the efficacy of chemotherapy

|  |  | **Efficacy (n)** ^a^ | | **Response** |  |  |
| --- | --- | --- | --- | --- | --- | --- |
| **SNP** | **Genotype** | **PD+SD** | **PR+CR** | **rate (%)** | **OR (95% CI)** ^b^ | ***P*-value** |
| rs174561 | T/T | 77 | 53 | 40.77 | Reference | 1.000 |
|  | C/T | 58 | 47 | 44.76 | 1.208 (0.720-2.024) | 0.512 |
|  | C/C | 17 | 22 | 56.41 | 1.905 (0.923-3.922) | 0.106 |
|  | C/T-C/C | 75 | 69 | 47.92 | 1.368 (0.850-2.203) | 0.226 |
|  | T/T-C/T | 135 | 100 | 42.55 | Reference | 1.000 |
|  | C/C | 17 | 22 | 56.41 | 1.748 (0.881-3.472) | 0.128 |
|  | T/T-C/C | 94 | 75 | 44.38 | Reference | 1.000 |
|  | C/T | 58 | 47 | 44.76 | 1.036 (0.637-1.689) | 0.902 |
| rs670637 | T/T | 146 | 117 | 44.49 | Reference | 1.000 |
|  | T/C | 6 | 5 | 45.45 | 1.138 (0.339-3.817) | 1.000 |
| rs2043556 | A/A | 67 | 52 | 43.70 | Reference | 1.000 |
|  | A/G | 75 | 65 | 46.43 | 1.129 (0.692-1.842) | 0.708 |
|  | G/G | 10 | 5 | 33.33 | 0.634 (0.212-1.894) | 0.585 |
|  | A/G-G/G | 85 | 70 | 45.16 | 1.068 (0.662-1.724) | 0.808 |
|  | A/A-A/G | 142 | 117 | 45.17 | Reference | 1.000 |
|  | G/G | 10 | 5 | 33.33 | 0.594 (0.205-1.715) | 0.428 |
|  | A/A-G/G | 77 | 57 | 42.54 | Reference | 1.000 |
|  | A/G | 75 | 65 | 46.43 | 1.188 (0.739-1.908) | 0.545 |
| rs2114358 | T/T | 76 | 68 | 47.22 | Reference | 1.000 |
|  | T/C | 61 | 40 | 39.60 | 0.733 (0.438-1.227) | 0.243 |
|  | C/C | 15 | 14 | 48.28 | 1.043 (0.469-2.320) | 1.000 |
|  | T/C-C/C | 76 | 54 | 41.54 | 0.794 (0.492-1.280) | 0.394 |
|  | T/T-T/C | 137 | 108 | 44.08 | Reference | 1.000 |
|  | C/C | 15 | 14 | 48.28 | 1.183 (0.548-2.558) | 0.696 |
|  | T/T-C/C | 91 | 82 | 47.40 | Reference | 1.000 |
|  | T/C | 61 | 40 | 39.60 | 0.728 (0.442-1.198) | 0.257 |
| rs2289030 | C/C | 103 | 72 | 41.14 | Reference | 1.000 |
|  | C/G | 42 | 47 | 52.81 | 1.575 (0.942-2.632) | 0.093 |
|  | G/G | 7 | 3 | 30.00 | 0.506 (0.123-2.088) | 0.498 |
|  | C/G-G/G | 49 | 50 | 50.51 | 1.420 (0.866-2.331) | 0.170 |
|  | C/C-C/G | 145 | 119 | 45.08 | Reference | 1.000 |
|  | G/G | 7 | 3 | 30.00 | 0.433 (0.106-1.767) | 0.314 |
|  | C/C-G/G | 110 | 75 | 40.54 | Reference | 1.000 |
|  | C/G | 42 | 47 | 52.81 | 1.626 (0.977-2.703) | 0.073 |
| rs2663345 | T/T | 33 | 32 | 49.23 | Reference | 1.000 |
|  | T/C | 95 | 65 | 40.63 | 0.706 (0.395-1.259) | 0.299 |
|  | C/C | 24 | 25 | 51.02 | 1.074 (0.512-2.257) | 1.000 |
|  | T/C-C/C | 119 | 90 | 43.06 | 0.780 (0.446-1.362) | 0.395 |
|  | T/T-T/C | 128 | 97 | 43.11 | Reference | 1.000 |
|  | C/C | 24 | 25 | 51.02 | 1.374 (0.740-2.551) | 0.343 |
|  | T/T-C/C | 57 | 57 | 50.00 | Reference | 1.000 |
|  | T/C | 95 | 65 | 40.63 | 0.684 (0.422-1.110) | 0.140 |
| rs4919510 | G/G | 52 | 41 | 44.09 | Reference | 1.000 |
|  | G/C | 78 | 69 | 46.94 | 1.122 (0.666-1.890) | 0.692 |
|  | C/C | 22 | 12 | 35.29 | 0.692 (0.307-1.560) | 0.421 |
|  | G/C-C/C | 100 | 81 | 44.75 | 1.028 (0.621-1.701) | 1.000 |
|  | G/G-G/C | 130 | 110 | 45.83 | Reference | 1.000 |
|  | C/C | 22 | 12 | 35.29 | 0.645 (0.305-1.362) | 0.273 |
|  | G/G-C/C | 74 | 53 | 41.73 | Reference | 1.000 |
|  | G/C | 78 | 69 | 46.94 | 1.235 (0.765-1.992) | 0.397 |
| rs9913045 | G/G | 93 | 82 | 46.86 | Reference | 1.000 |
|  | G/A | 49 | 34 | 40.96 | 0.787 (0.464-1.335) | 0.422 |
|  | A/A | 10 | 6 | 37.50 | 0.680 (0.237-1.953) | 0.603 |
|  | G/A-A/A | 59 | 40 | 40.40 | 0.769 (0.467-1.267) | 0.314 |
|  | G/G-G/A | 142 | 116 | 44.96 | Reference | 1.000 |
|  | A/A | 10 | 6 | 37.50 | 0.734 (0.259-2.079) | 0.614 |
|  | G/G-A/A | 103 | 88 | 46.07 | Reference | 1.000 |
|  | G/A | 49 | 34 | 40.96 | 0.812 (0.482-1.368) | 0.509 |
| rs10061133 | A/A | 87 | 75 | 46.30 | Reference | 1.000 |
|  | G/A | 58 | 39 | 40.21 | 0.780 (0.468-1.299) | 0.367 |
|  | G/G | 7 | 8 | 53.33 | 1.326 (0.459-3.831) | 0.788 |
|  | G/A-G/G | 65 | 47 | 41.96 | 0.839 (0.516-1.364) | 0.537 |
|  | A/A-G/A | 145 | 114 | 44.02 | Reference | 1.000 |
|  | G/G | 7 | 8 | 53.33 | 1.453 (0.512-4.132) | 0.595 |
|  | A/A-G/G | 94 | 83 | 46.89 | Reference | 1.000 |
|  | G/A | 58 | 39 | 40.21 | 0.762 (0.461-1.258) | 0.311 |
| rs11614913 | T/T | 40 | 38 | 48.72 | Reference | 1.000 |
|  | C/T | 80 | 56 | 41.18 | 0.739 (0.422-1.295) | 0.321 |
|  | C/C | 32 | 28 | 46.67 | 0.911 (0.464-1.786) | 0.864 |
|  | C/T-C/C | 112 | 84 | 42.86 | 0.789 (0.465-1.337) | 0.422 |
|  | T/T-C/T | 120 | 94 | 43.93 | Reference | 1.000 |
|  | C/C | 32 | 28 | 46.67 | 1.104 (0.623-1.953) | 0.773 |
|  | T/T-C/C | 72 | 66 | 47.83 | Reference | 1.000 |
|  | C/T | 80 | 56 | 41.18 | 0.770 (0.479-1.239) | 0.333 |
| rs13299349 | G/G | 119 | 87 | 42.23 | Reference | 1.000 |
|  | G/A | 33 | 33 | 50.00 | 1.366 (0.782-2.387) | 0.324 |
|  | A/A | 0 | 2 | 100.0 | 1.981 (1.729-2.269) | 0.247 |
|  | G/A-A/A | 33 | 35 | 51.47 | 1.471 (0.847-2.551) | 0.212 |
|  | G/G-G/A | 152 | 120 | 44.12 | Reference | 1.000 |
|  | A/A | 0 | 2 | 100.0 | 1.908 (1.704-2.138) | 0.250 |
|  | G/G-A/A | 119 | 89 | 42.79 | Reference | 1.000 |
|  | G/A | 33 | 33 | 50.00 | 1.328 (0.760-2.315) | 0.329 |
| rs35770269 | A/A | 43 | 40 | 48.19 | Reference | 1.000 |
|  | A/T | 84 | 62 | 42.47 | 0.794 (0.462-1.364) | 0.410 |
|  | T/T | 25 | 20 | 44.44 | 0.860 (0.415-1.783) | 0.714 |
|  | A/T-T/T | 109 | 82 | 42.93 | 0.808 (0.482-0.576) | 0.431 |
|  | A/A-A/T | 127 | 102 | 44.54 | Reference | 1.000 |
|  | T/T | 25 | 20 | 44.44 | 0.996 (0.524-1.894) | 1.000 |
|  | A/A-T/T | 68 | 60 | 46.88 | Reference | 1.000 |
|  | A/T | 84 | 62 | 42.47 | 0.837 (0.519-1.350) | 0.468 |
| rs61992671 | A/A | 150 | 115 | 43.40 | Reference | 1.000 |
|  | A/G | 2 | 6 | 75.00 | 3.559 (0.725-17.54) | 0.173 |
|  | G/G | 0 | 1 | 100.0 | 2.015 (1.785-2.276) | 0.498 |
|  | A/G-G/G | 2 | 7 | 77.78 | 4.065 (0.847-19.61) | 0.103 |
|  | A/A-A/G | 152 | 121 | 44.32 | Reference | 1.000 |
|  | G/G | 0 | 1 | 100.0 | 1.978 (1.759-2.225) | 1.000 |
|  | A/A-G/G | 150 | 116 | 43.61 | Reference | 1.000 |
|  | A/G | 2 | 6 | 75.00 | 3.521 (0.719-17.24) | 0.173 |
| rs67106263 | G/G | 110 | 91 | 45.27 | Reference | 1.000 |
|  | G/A | 40 | 28 | 41.18 | 0.846 (0.488-1.468) | 0.577 |
|  | A/A | 2 | 3 | 60.00 | 1.515 (0.248-9.259) | 1.000 |
|  | G/A-A/A | 42 | 31 | 42.47 | 0.880 (0.515-1.506) | 0.683 |
|  | G/G-G/A | 150 | 119 | 44.24 | Reference | 1.000 |
|  | A/A | 2 | 3 | 60.00 | 1.580 (0.260-9.615) | 0.679 |
|  | G/G-A/A | 112 | 94 | 45.63 | Reference | 1.000 |
|  | G/A | 40 | 28 | 41.18 | 0.838 (0.484-1.451) | 0.577 |
| rs73239138 | G/G | 74 | 49 | 39.84 | Reference | 1.000 |
|  | G/A | 49 | 51 | 51.00 | 1.572 (0.923-2.681) | 0.106 |
|  | A/A | 29 | 22 | 43.14 | 1.145 (0.591-2.217) | 0.736 |
|  | G/A-A/A | 78 | 73 | 48.34 | 1.412 (0.873-2.288) | 0.180 |
|  | G/G-G/A | 123 | 100 | 44.84 | Reference | 1.000 |
|  | A/A | 29 | 22 | 43.14 | 0.933 (0.505-1.724) | 0.877 |
|  | G/G-A/A | 103 | 71 | 40.80 | Reference | 1.000 |
|  | G/A | 49 | 51 | 51.00 | 1.511 (0.920-2.475) | 0.130 |

^a^ PD, progressive disease; SD, stable disease; PR, partial response; CR, complete response.

^b^ OR, odds ratio; CI, confidence interval.
