# Supplementary material for: The SNPs in pre-miRNA are related to the response of capecitabine-based therapy in advanced colon cancer patients
Source: Oncotarget. 2017 Dec 11;9(6):6793–9. doi: 10.18632/oncotarget.23190 (PMC5805515; doi:10.18632/oncotarget.23190)
Supplement: Supplementary file 4 [file oncotarget-09-6793-s004.docx]

**Supplementary Table 3** The association of genotypes with the side effects of chemotherapy

|  |  | **Side effect, n** | | **Incidence** |  |  |
| --- | --- | --- | --- | --- | --- | --- |
| **SNP** | **Genotype** | **No** | **Yes** | **(%)** | **OR (95% CI)**^a^ | ***P*-value** |
| rs174561 | T/T | 38 | 92 | 70.77 | Reference | 1.000 |
|  | C/T | 36 | 69 | 65.71 | 0.737 (0.437-1.242) | 0.288 |
|  | C/C | 12 | 27 | 69.23 | 0.877 (0.432-1.779) | 0.721 |
|  | C/T-C/C | 48 | 96 | 66.67 | 0.774 (0.478-1.253) | 0.328 |
|  | T/T-C/T | 74 | 161 | 68.51 | Reference | 1.000 |
|  | C/C | 12 | 27 | 69.23 | 1.010 (0.520-1.961) | 1.000 |
|  | T/T-C/C | 50 | 119 | 70.41 | Reference | 1.000 |
|  | C/T | 36 | 69 | 65.71 | 0.762 (0.466-1.244) | 0.317 |
| rs670637 | T/T | 83 | 180 | 68.44 | Reference | 1.000 |
|  | T/C | 3 | 8 | 72.73 | 1.087 (0.324-3.650) | 1.000 |
| rs744591 | A/A | 27 | 62 | 69.66 | Reference | 1.000 |
|  | A/C | 43 | 84 | 66.14 | 0.801 (0.462-1.387) | 0.486 |
|  | C/C | 16 | 42 | 72.41 | 1.190 (0.608-2.331) | 0.733 |
|  | A/C-C/C | 59 | 126 | 68.11 | 0.907 (0.543-1.517) | 0.794 |
|  | A/A-A/C | 70 | 146 | 67.59 | Reference | 1.000 |
|  | C/C | 16 | 42 | 72.41 | 1.359 (0.756-2.439) | 0.378 |
|  | A/A-C/C | 43 | 104 | 70.75 | Reference | 1.000 |
|  | A/C | 43 | 84 | 66.14 | 0.746 (0.462-1.205) | 0.272 |
| rs745666 | G/G | 21 | 59 | 73.75 | Reference | 1.000 |
|  | G/C | 52 | 87 | 62.59 | 0.712 (0.410-1.238) | 0.263 |
|  | C/C | 13 | 42 | 76.36 | 1.490 (0.737-3.012) | 0.293 |
|  | G/C-C/C | 65 | 129 | 66.49 | 0.880 (0.522-1.484) | 0.691 |
|  | G/G-G/C | 73 | 146 | 66.67 | Reference | 1.000 |
|  | C/C | 13 | 42 | 76.36 | 1.842 (0.998-3.390) | 0.053 |
|  | G/G-C/C | 34 | 101 | 74.81 | Reference | 1.000 |
|  | G/C | 52 | 87 | 62.59 | 0.608 (0.376-0.982) | 0.052 |
| rs2043556 | A/A | 38 | 81 | 68.07 | Reference | 1.000 |
|  | A/G | 42 | 98 | 70.00 | 1.094 (0.645-1.855) | 0.788 |
|  | G/G | 6 | 9 | 60.00 | 0.704 (0.234-2.119) | 0.566 |
|  | A/G-G/G | 48 | 107 | 69.03 | 1.046 (0.625-1.748) | 0.896 |
|  | A/A-A/G | 80 | 179 | 69.11 | Reference | 1.000 |
|  | G/G | 6 | 9 | 60.00 | 0.670 (0.231-1.946) | 0.568 |
|  | A/A-G/G | 44 | 90 | 67.16 | Reference | 1.000 |
|  | A/G | 42 | 98 | 70.00 | 1.140 (0.684-1.901) | 0.696 |
| rs2289030 | C/C | 59 | 116 | 66.29 | Reference | 1.000 |
|  | C/G | 23 | 66 | 74.16 | 1.460 (0.826-2.577) | 0.208 |
|  | G/G | 4 | 6 | 60.00 | 0.763 (0.207-2.809) | 0.737 |
|  | C/G-G/G | 27 | 72 | 72.73 | 1.357 (0.789-2.331) | 0.282 |
|  | C/C-C/G | 82 | 182 | 68.94 | Reference | 1.000 |
|  | G/G | 4 | 6 | 60.00 | 0.676 (0.186-2.457) | 0.511 |
|  | C/C-G/G | 63 | 122 | 65.95 | Reference | 1.000 |
|  | C/G | 23 | 66 | 74.16 | 1.481 (0.843-2.604) | 0.211 |
| rs2663345 | T/T | 17 | 48 | 73.85 | Reference | 1.000 |
|  | T/C | 55 | 105 | 65.63 | 0.664 (0.362-1.220) | 0.226 |
|  | C/C | 14 | 35 | 71.43 | 0.775 (0.364-1.647) | 0.566 |
|  | T/C-C/C | 69 | 140 | 66.99 | 0.691 (0.384-1.241) | 0.244 |
|  | T/T-T/C | 72 | 153 | 68.00 | Reference | 1.000 |
|  | C/C | 14 | 35 | 71.43 | 1.043 (0.568-1.916) | 1.000 |
|  | T/T-C/C | 31 | 83 | 72.81 | Reference | 1.000 |
|  | T/C | 55 | 105 | 65.63 | 0.749 (0.459-1.221) | 0.266 |
| rs4919510 | G/G | 27 | 66 | 70.97 | Reference | 1.000 |
|  | G/C | 47 | 100 | 68.03 | 0.893 (0.527-1.513) | 0.690 |
|  | C/C | 12 | 22 | 64.71 | 0.564 (0.248-1.284) | 0.210 |
|  | G/C-C/C | 59 | 122 | 67.40 | 0.826 (0.496-1.374) | 0.519 |
|  | G/G-G/C | 74 | 166 | 69.17 | Reference | 1.000 |
|  | C/C | 12 | 22 | 64.71 | 0.606 (0.286-1.285) | 0.249 |
|  | G/G-C/C | 39 | 88 | 69.29 | Reference | 1.000 |
|  | G/C | 47 | 100 | 68.03 | 1.035 (0.640-1.672) | 0.903 |
| rs9913045 | G/G | 50 | 125 | 71.43 | Reference | 1.000 |
|  | G/A | 31 | 52 | 62.65 | 0.877 (0.517-1.490) | 0.685 |
|  | A/A | 5 | 11 | 68.75 | 1.133 (0.387-3.311) | 1.000 |
|  | G/A-A/A | 36 | 63 | 63.64 | 0.913 (0.553-1.506) | 0.798 |
|  | G/G-G/A | 81 | 177 | 68.60 | Reference | 1.000 |
|  | A/A | 5 | 11 | 68.75 | 1.179 (0.408-3.413) | 0.797 |
|  | G/G-A/A | 55 | 136 | 71.20 | Reference | 1.000 |
|  | G/A | 31 | 52 | 62.65 | 0.869 (0.515-1.466) | 0.688 |
| rs10061133 | A/A | 48 | 114 | 70.37 | Reference | 1.000 |
|  | G/A | 32 | 65 | 67.01 | 0.855 (0.498-1.468) | 0.581 |
|  | G/G | 6 | 9 | 60.00 | 0.632 (0.213-1.873) | 0.395 |
|  | G/A-G/G | 38 | 74 | 66.07 | 0.820 (0.489-1.374) | 0.508 |
|  | A/A-G/A | 80 | 179 | 69.11 | Reference | 1.000 |
|  | G/G | 6 | 9 | 60.00 | 0.670 (0.231-1.946) | 0.568 |
|  | A/A-G/G | 54 | 123 | 69.49 | Reference | 1.000 |
|  | G/A | 32 | 65 | 67.01 | 0.892 (0.525-1.515) | 0.685 |
| rs11614913 | T/T | 27 | 51 | 65.38 | Reference | 1.000 |
|  | C/T | 41 | 95 | 69.85 | 1.193 (0.680-2.096) | 0.567 |
|  | C/C | 18 | 42 | 70.00 | 1.041 (0.533-2.033) | 1.000 |
|  | C/T-C/C | 59 | 137 | 69.90 | 1.143 (0.674-1.938) | 0.686 |
|  | T/T-C/T | 68 | 146 | 68.22 | Reference | 1.000 |
|  | C/C | 18 | 42 | 70.00 | 0.931 (0.527-1.645) | 0.884 |
|  | T/T-C/C | 45 | 93 | 67.39 | Reference | 1.000 |
|  | C/T | 41 | 95 | 69.85 | 1.172 (0.727-1.890) | 0.544 |
| rs13299349 | G/G | 64 | 142 | 68.93 | Reference | 1.000 |
|  | G/A | 22 | 44 | 66.67 | 0.902 (0.499-1.629) | 0.762 |
|  | A/A | 0 | 2 | 100.0 | 1.451 (1.324-1.590) | 1.000 |
|  | G/A-A/A | 22 | 46 | 67.65 | 0.943 (0.524-1.695) | 0.881 |
|  | G/G-G/A | 86 | 186 | 68.38 | Reference | 1.000 |
|  | A/A | 0 | 2 | 100.0 | 1.462 (1.349-1.585) | 1.000 |
|  | G/G-A/A | 64 | 144 | 69.23 | Reference | 1.000 |
|  | G/A | 22 | 44 | 66.67 | 0.889 (0.493-1.605) | 0.761 |
| rs61992671 | A/A | 83 | 182 | 68.68 | Reference | 1.000 |
|  | A/G | 3 | 5 | 62.50 | 0.508 (0.119-2.169) | 0.478 |
|  | G/G | 0 | 1 | 100.0 | 1.846 (1.652-2.063) | 0.502 |
|  | A/G-G/G | 3 | 6 | 66.67 | 0.846 (0.239-2.994) | 1.000 |
|  | A/A-A/G | 86 | 187 | 68.50 | Reference | 1.000 |
|  | G/G | 0 | 1 | 100.0 | 1.863 (1.668-2.080) | 0.501 |
|  | A/A-G/G | 83 | 183 | 68.80 | Reference | 1.000 |
|  | A/G | 3 | 5 | 62.50 | 0.501 (0.117-2.137) | 0.477 |
| rs67106263 | G/G | 62 | 139 | 69.15 | Reference | 1.000 |
|  | G/A | 23 | 45 | 66.18 | 0.842 (0.489-1.453) | 0.579 |
|  | A/A | 1 | 4 | 80.00 | 1.264 (0.207-7.752) | 1.000 |
|  | G/A-A/A | 24 | 49 | 67.12 | 0.865 (0.508-1.473) | 0.684 |
|  | G/G-G/A | 85 | 184 | 68.40 | Reference | 1.000 |
|  | A/A | 1 | 4 | 80.00 | 1.321 (0.217-8.065) | 1.000 |
|  | G/G-A/A | 63 | 143 | 69.42 | Reference | 1.000 |
|  | G/A | 23 | 45 | 66.18 | 0.838 (0.487-1.443) | 0.579 |

^a^ OR, odds ratio; CI, confidence interval.
